# Book Reviews

**Published:** 1812-05

**Authors:** 


### CRITICAL ANALYSIS

### OF RECENT PUBLICATIONS

IN THE

### DIFFERENT BRANCHES OF PHYSIC, SURGERY, AND MEDICAL PHILOSOPHY.

*A Collection of Treatises on the Effects of Sol-lunar Influence in Fevers; with an improved Method of curing them. By* FRANCIS BALFOUR, M. D. First Member of the Medical Board in Bengal. Second edition. 8vo. pp. 359. 1811.

FROM the earliest annals of medicine, it appears that the heavenly bodies have been supposed to have considerable influence upon vegetable and animal creation. It accorded accorded with the superstition of the dark ages, and was supported by the testimony of philosophers in more enlightened periods. The ebbing and flowing of the tide, certain changes of atmosphere, currents, winds, &c. are generally admitted to be affected by this influence. In ancient writers we find instructions for gathering herbs under particular states of the moon; the character of man is supposed by many to be determined by the aspect of the planet under which he is born; and at this day many very respectable practitioners believe that the moon regulates the periodical flow of the catamenia, affects maniacs, and occasions epileptic fits. Unprejudiced experience, however, opposes the supposition; without disputing the theory of tides, we may be allowed to deny that the states of the moon have any influence upon the diseases just enumerated. The most intelligent writer upon madness, Mr. Haslam, whose opportunities for observation are not greater than his power of adapting them to professional information, tells us, that he kept a register for two years, but without finding, *in any instance*, that the aberrations of human intellect corresponded with, or were influenced by, the vicissitudes of the moon. Every day's experience proves that it has no influence on the catamenia; and the recurrence of epileptic fits seems to be no more affected by it than the succession of paroxysms in an ague.

Dr. Mead wrote a very learned treatise on the subject, but he by no means established the certainty of sol-lunar influence upon diseases. Where the cause is universal, surely the effect should not be so sparing and obscure, as to escape the notice of the most acute and intelligent observers of nature. Dr. Balfour, however, to whom it becomes us now to pay some attention, is fully convinced of this influence, and has reasoned upon it with mathematical precision. In the present work, he chiefly considers its operation on fevers. As he states facts which came under his immediate observation, we shall present our readers with so much of them as may enable them to judge of the importance of his discoveries.

In his first treatise he sets out with certain propositions, which he supports argumentatively.

---

sion of Mr. Leschenault, when I had the pleasure of knowing this intelligent botanist, and of inspecting a part of his rich collection of plants, &c. in Philadelphia, in 1807. The *Aquarium* grows in a moist but firm soil. Its radical leaves are hollowed, and are in shape somewhat like a water-pot. Each pot is about an inch long, and is capable of containing a good deal of water.

"PROPOSITION

"PROPOSITION I.—*In Bengal, fevers of every denomination are, in a remarkable manner, connected with, and affected by, the revolutions of the moon.*—The bilious intermittent fever, which appears for the most part in the form of a tertian, or of a quotidian, and seldom in that of a quartan, is by far the most common fever in this country. In whatever form it presents itself, I have almost invariably observed that its first attack is on one of the three days which immediately precede the full of the moon; or one of the three days which immediately precede and follow the change of the moon. I have observed the remarkable connection which prevails at this time, evidently at least three complete days both before and after the full and change of the moon; so that it continues at least six complete days at each. In general, I think, that the days of the full and change are more powerful than any other, and those that follow the full and change more powerful than those that precede; but my observations respecting this point do not allow me to speak with any confidence.

"The full and change of the moon are no less remarkable for occasioning relapses than for inducing the first attack of bilious fevers. This is a fact so well established, that there are few Europeans, who have resided for any time in this country, who are not sufficiently informed of it, either from their own personal experience, or from the daily proofs of it that occur in the circle of their acquaintances. But it cannot possibly escape the notice of any person who practises medicine with the smallest attention for a few months. For my own part I have observed this tendency to relapse at the full and change invariably for these fourteen years; and in particular cases can prognosticate the return of the fever, at these periods, with almost as much confidence as I can foretel the revolution itself."

The doctor does not confine this influence to intermittents and remittents alone, for he also remarks that,

"Head-achs, tooth-achs, inflammations of the eyes, asthmas, pains and swelling of the liver and spleen, fluxes, spasms and obstructions in the bowels, complaints in the urinary passages, eruptions of different kinds, and a great many more unattended by any obvious fever, assume often an intermitting form; and regularly return or increase with the full and change of the moon; and disappear and diminish during the intervals."

"PROPOSITION II.—*In Bengal, a constant and particular attention to the revolutions of the moon, is of the greatest importance in the cure and prevention of fevers.*"

The author illustrates this proposition, by stating the effects of Peruvian bark in the cure of these fevers; he prescribed it very abundantly, and always with good success; taking care, however, to clear the stomach and bowels of bile, by evacuants. He thus applies the facts, which he considers he has established respecting the influence of the moon, in the cure of fevers.

"1st.

"1st. When an intermittent of any kind appears towards the end of the intervals, the first object to be held in view is to put a stop, if possible, before the approach of the full and change: because, as I have already observed, the paroxyms then become more severe, of longer duration, and more difficult to cure; and will sometimes continue so long as to run into one another, and assume the form of a remittent; and afford no convenient opportunity for exhibiting the bark during the whole of that period. And, although evacuations alone will generally remove the fever in the intervals, this is scarcely to be expected during the full and change. For the same reason, when intermittents appear at the beginning of the full or change, the same object must be held in view; otherwise we must not look for a solution of the fever till that period be at an end.

"2d. On the other hand it is to be observed, that, when intermittents make their appearance towards the end of the full and change, there is not the same occasion for a hasty exhibition of the bark: because there is a probability, if not of a spontaneous solution of the fever, at least of an abatement of its severity upon the expiration of these periods.

"3d. One of the most important advantages to be derived from an attention to this system, is the mode suggested by it of securing against relapses. These generally happen at the full and change, and no person who has had an intermittent, can consider himself in any degree safe at these periods, until he has perfectly recovered his strength, and removed every symptom of obstruction. It is therefore absolutely necessary to watch these returns with the greatest care; and in general the use of laxatives, and a few doses of bark given a day before, and continued every day whilst the period lasts, will prevent a relapse. When these precautions prove ineffectual, and the patient, in spite of all his endeavors, neither recovers strength, nor gets quit of the symptoms of obstructions, we are then taught to remove him with all expedition to a climate where the influence of the moon is less perceivable, and less prejudicial, than it is in Bengal.*

"4th. With respect to bilious remittents, they are to be considered as no other than quotidian and tertian intermittents, whose fits are protracted by bile retained in the bowels, or taken up into the circulation, by obstructions of the liver and spleen, by the influence of the moon, or some other cause; and in them an attention to all the different circumstances, we have just now pointed out, is still more necessary than in intermittents; in proportion as their progress is more rapid, their danger greater, and their management more difficult.

"5th. Putrid, nervous, and rheumatic, fevers are all in this country (Bengal,) equally under the influence of the moon; and in all our attention to these observations will be of the greatest use, both in treating them, when present, and in preventing relapses."

* At Madras it is much less felt, and a removal to that settlement from Bengal is, in many cases, almost a certain cure.

The author's experience also convinced him that the eruption and fever in small-pox were materially affected by the sol-lunar influence; and he applied this knowledge advantageously, by inoculating on the second or third day of the full and change, so that the eruptive fever might occur in the intervals.

He farther affirms, that the cure of head-achs, tooth-achs, opthalmia, asthma, pains and swellings of the liver and spleen, fluxes, spasms, obstructions in the bowels, complaints in the urinary passages, eruptions of various kinds, &c. &c. which return periodically with the moon, whether attended with fever or not, "entirely depends on a constant attention to these revolutions."

"By every succeeding return of such complaints, the parts affected grow weaker and weaker, more liable to relapse, and more difficult to cure. On the other hand, by preventing each return, the parts have a longer interval for gaining strength, become less subject to relapses, and at last recover their former tone. Therefore, when such complaints do not originate from a diseased liver, a proper attention to regimen and to the state of the bowels, a judicious derivation from the part affected, and a timely exhibition of the bark before the approach of the lunar revolution, and during their continuation, will in general succeed. But it is to be remembered, that such periodical complaints, in almost every case, are connected with a diseased liver, which is best cured by mercury; and the bark is nevertheless to be given at the full and change in such quantity as to prevent relapses."

'PROPOSITION III.—*The influence of the moon in fevers prevails in a similar manner in every inhabited quarter of the globe; and consequently a similar attention to it is a matter of general importance in the practice of medicine.*'

The author entirely fails in proving this proposition; which, if true, would not have obliged him to have recourse to reasoning from analogy. In another part of his work, indeed, he supports it by his observations in Scotland, but the evidence he has adduced is neither convincing nor satisfactory. Surely the influence of the sun or moon upon fevers would not have entirely escaped the notice of practitioners, who, in severe cases, watch the progress of the complaint great part of the day and night, without ever observing the crises and changes alluded to by the learned doctor.

In the fourth proposition he asserts, that "*The whole doctrine of the crisis of fevers may be easily explained from the premises we have established, respecting these disorders at the full and change.*"—Here, too, the author is obliged to rest upon his own experience, and even cannot derive assistance from those writers who have more especially insisted upon the doctrine of crises; for they, it seems, not thinking of the sun sun or the moon, neglected to afford the necessary dates by which it might be made evident, that the remission and paroxysms of the disorder were simultaneous with certain changes of those luminaries.

The second treatise is upon Putrid intestinal Remitting Fevers; and contains much ingenious reasoning, and many interesting facts respecting those complaints; from which the author establishes certain axioms respecting the laws of sol-lunar influence.

In the third treatise, *on the Action of Sol-lunar Influence*, he exhibits by a minute analysis of these axioms, the first and most simple phenomena with which his observations originated.

In the fourth treatise, this influence in fevers is considered, and illustrated by numerous facts from practitioners in various quarters. The fifth treatise is upon the Barometer; and the sixth, which concludes the volume, treats "*of Sol-lunar Influence in the Fevers of India.*"

The subject of this volume is certainly curious, and we hope that it may pass into the hands of men whose talents and opportunities may enable them to continue an investigation which hitherto has obtained little encouragement or success. Dr. Balfour most strenuously advocates the cause he has engaged in, and, whether we admit or deny his positions, he merits the praise of great ingenuity, perseverance, and industry.

---

*Annales de Chimie, ou Recueil de Memoires concernant la Chimie et les Arts qui en dependent, et specialement la Pharmacie*, &c. tom. 77. Paris. 1811.

In noticing this valuable collection of chemical facts, our limits necessarily oblige us to select those which most immediately concern our profession, or which are especially curious and interesting.

The volume commences with some observations upon the simultaneous progression of mechanical coherence and chemical affinity; by M. Ermann, professor and member of the Academy of Sciences of Berlin. The memoir contains an account of several experiments, on which the ingenious professor has founded the following conclusions.

As soon as galvanism excites chemical affinities, the intensity of the attraction of the surfaces is augmented. This confirms the analogy previously supposed, between adhesion and chemical affinity.

Electricity determines a greater attraction between bodies which act chemically on each other. But this attraction is wholly distinct from the electric attraction hitherto known.

 There

There is reason to presume that, in the galvanic process, attraction at a sensible distance, and attraction of surfaces, act in concert. The augmented attraction of surfaces, and that of particles taking place in determined polar points, would then be the immediate physical product: the chemical product would be reunited to it by the bond which exists between chemical affinity and cohesion.

From a memoir upon the extraction of sugar from beet-root, by M. Deyeux, we find that he was successful, and that a manufactory on a large scale has been instituted to supply the urgent demand for sugar in France. From the difficulty of the process, and the small quantity of sugar obtained, we do not expect that the speculation will prove very advantageous. The attempts to extract this necessary article from raisins and other vegetable productions have failed; the small quantity of saccharine matter produced has not been of good quality, and would not admit of the process of refining sufficiently to become perfect sugar.

In a note upon Prussic acid, read before the Institute in February 1811, M. Gay Lussac observes, that, since the discovery of Prussic acid by Scheele, and the labors of Berthollet and Clouet, nothing of importance on the nature of this acid, has been added. It has not yet been obtained perfectly pure, and we are ignorant how it would appear in that state. M. Gay Lussac has therefore directed his attention to the subject, and has endeavoured to prove that Prussic acid does not possess permanent elasticity; and that it forms a liquid much more volatile than sulphuric ether. Desirous to ascertain by experiments if he could obtain Prussic acid in a gaseous state, he decomposed Prussiat of mercury by muriatic acid, as indicated by M. Proust. After having permitted the air to escape from the glass bells, and perceiving a very strong odour of Prussic acid, he received the gases on mercury; and thus obtained an elastic, inflammable, strong-smelling fluid, which appeared to him to be gaseous Prussic acid. But, in continuing the operation, he perceived that some drops of a peculiar fluid assumed the gaseous state as soon as they arrived at the top of the glass bell, and strongly depressed the column of mercury. The temperature was then 20° *centigrades,* and the following day, being only 12°, he observed that the gaseous volume which he had obtained was much diminished, and that a fluid was deposited in the glass vessels, where there was none before.

M. Gay Lussac now put some Prussiat of mercury into a tubulated retort, to the beak of which he adapted a bent tube, one of the branches of which he plunged in a small tubulated bottle, containing chalk and muriate of lime; the chalk being intended to saturate the muriatic acid which might might escape from the retort, and the muriate of lime to retain the water. From this bottle another tube passed into a second tubulated bottle, containing muriate of lime; and from this, a third tube passed into a small bottle stopped with emery, and destined to receive the Prussic acid.

The apparatus being thus disposed, and all the bottles surrounded with a cooling mixture, of two parts of ice and one of salt, the experimenter poured muriatic acid into the retort, and applied a gentle heat. The Prussiat of mercury was soon dissolved, and the liquor appeared in a state of ebullition. Vapors were disengaged and condensed, in part, in the neck of the retort. The operation was stopped the moment the water began to evaporate: Prussic acid might still be obtained, but it is better to separate the first product, and then resume the distillation.

All the Prussic acid is usually condensed in the first bottle. If no water has passed, the muriate of lime remains solid, though washed by the Prussic acid. If, on the contrary, a certain quantity has passed, two very distinct liquids will be obtained; the lowest is an aqueous solution of muriate of lime, and the uppermost is Prussic acid. This acid is generally a little colored in the first bottle. To rectify it, the tube communicating with the retort must be removed as soon as it is intended to terminate the distillation; the opening through which the tube entered the bottle must be closed; and, after having taken away the cooling mixture which surrounded it, a gentle heat is to be applied. When the distillation is finished, the first bottle is to be removed; and, after the Prussic acid has remained some hours in contact with the muriate of lime in the second bottle, it should be made to pass into the third by a gentle heat; and then the rectification is completed.

The Prussic acid thus obtained is a limpid colorless liquid like water. Its savour at first cool, soon becomes pungent and irritating. Though rectified several times upon chalk, it feebly reddens paper tinged with turnsole: the blue color reappears as soon as the acid evaporates. Its density at $7° = 0{,}70583$. It is extremely volatile; subjected to a cooling mixture of two parts of ice and one of salt, it congeals and often takes a regular form.

A memoir upon the triple salts, by the same author,* was read before the "Société d'Arcueil," the 10th of February, 1811; in which he attempts to prove,

* This memoir will be published in the third volume of the "*Memoires de la Société d'Arcueil.*"

1. That

1. That, in the triple salts, the acid commonly separates itself into two equal parts between the two lenses.

2. That, in a triple combination, the elements, reunited two by two, form possible binary compounds. For example, nitrate of ammonia, which is composed of oxygen, azote, and hydrogen, being decomposed by heat, gives rise to water, and gaseous oxide of azote; whilst, on the other hand, this salt is the result of two binary compounds, nitric acid and ammonia.

3. That vegetable and animal substances, which are composed of three or four different substances, also give rise to possible binary compounds, or known in general.

4. That we may conceive the different nature of several bodies containing the same elements, and in the same proportions; by admitting that the binary products of the elements combine together in different ways between them, or only with one of the elements.

5. That we may conceive as many more compounds containing the same elements, in equal quantity, as we can conceive more possible binary compounds, formed by the elements of those same compounds.

6. That, salts, or other compounds, being neutral, though formed by an acid which contains an excess of oxygen, and a base which is still combustible; we may admit that the base saturates the excess of oxygen of the acid, and that a point of saturation results from it, very proper to determine the capacity of combustibles for oxygen.

7. That nitrous gas and oxygen gas, in combining to produce nitrous acid gas, experience an apparent condensation of volume which is exactly half of the total volume of the two gases; from which it results that the density of the nitrous acid gas = 2,10633; that of the air being taken for unity.

---

In a Dissertation "*upon the Changes which the Eggs and Larvæ of certain Insects effect upon the Physical and Medicinal Properties of the Flowers of the Arnica Montana, (Lin.")* M. F. M. Mercier, M. D. remarks, that, as the flowers of the Arnica Montana are frequently employed in medicine, it may not be useless to observe, that the choice of them is not a matter of indifference. It is of consequence to take care that they are not soiled by the eggs and larvæ of the insects which frequent them; it is of consequence to know the changes which the presence of these eggs and larvæ makes them undergo; in short, to distinguish readily those which are exempt from these impurities, and those which are not.

Dr.

Dr. Mercier informs us, that several circumstances had induced him to appreciate the medicinal properties of the flowers of the arnica. He remarked a considerable difference in the effects of an infusion of these flowers, in cases nearly similar. Some individuals affected with asthenic diseases, with a soft fibre, and dull sensibility, complained of a troublesome sensation of heat in the throat and stomach; cardialgia, nausea, and vomiting, each time after taking an infusion of the dried flowers of arnica, in the dose of fifteen grains to a quart of water. The same dose of the flowers, gathered by other hands, and a double dose of the root, boiled in the same quantity of water, were not followed by these unpleasant symptoms.

It seemed probable then that some extraordinary adulteration had occurred with the flowers first used. Upon examining them minutely, Dr. Mercier found them filled with small, black, dirty, oval, shells, from one to two millimètres* long, somewhat resembling the dung of mice. Some of them were bruised, others empty, and pierced through one of their extremities; several were whole, and served as a covering to a dry matter, of a yellowish white, which, pressed between the fingers, crumbled into powder. The flowers in which these little bodies were concealed, separately considered, had lost the beautiful yellow and peculiar aroma which distinguish them. The florets were lost in an agglutinated greyish mass, which covered the receptacle and the calyx. In the interior of these, and the spaces between them, were contained the small shells. Upon removing the whole, the receptacle in some was found entire, in others it was eaten.

The ingenious author subjected them to various tests, and, after many experiments, concluded, that boiling water, in imbibing the properties of the flowers of arnica, also becomes charged with a portion of those belonging to the heterogeneous bodies which they enclose. From these the infusion derives, in part, the property of irritating the throat and the stomach, and produces the cardialgia, nausea, vomiting, &c. Further, these bodies, which are the eggs of certain insects, leave the flowers, from which they are extracted, impregnated with their acrid quality; whether it is communicated by the insects themselves, by the viscous humors with which they were enveloped, or by the larvæ which proceeded from them. All such flowers should be carefully rejected, as they are hardly less pernicious than those which have not been cleansed from the eggs.

---

* The French term is *litre,* a measure containing nearly $2\frac{1}{8}$ wine pints.—Millimetre =,03937 E. inches.

The

The distinction between the pure flowers, and those which are spoiled, is observed wherever the plant grows, whether on mountains, or low lands, in pasture grounds, or meadows in vallies; in the north or the south, the east or the west. The pure flowers are characterised by their beautiful yellow color, aroma, and freshness: they have not been soiled by any foreign matter. The florets are very distinct, and have a brilliant, saffron-colored, appearance.

The spoiled flowers, which conceal the eggs of the insects, appear faded and withered, are discolored, and exhale little or no smell. The buds are pale and pendant; the flowrets, greyish and agglutinated together, form a kind of covering, which serves as a shelter for the eggs and larvæ enclosed in the interior, or in the space between the small calices. The eggs were chiefly black, some of a yellow white; all of them, in size and shape, resembled ants eggs, though rather smaller.

In some of the oldest and most withered flowers, the larvæ were observed half disengaged from the eggs, eating a seed, or endeavouring to force themselves deeper towards the receptacle.

All the larvæ were apodous, or their feet were only marked on each side by projecting points. Their body was soft, of a yellow-white, and from five to six millimetres long. A black spot was observed on each side the head. Dr. Mercier could not determine to what insects the eggs and larvæ belonged. They were not peculiar to the arnica, for he also found them on other flowers, as those of *inula dyssenterica, doronicum pardalianches, conyza squarrosa, artemisia rupestris, &c.*

As the physician expects, when he prescribes a remedy, that it should be in a proper condition for use, the particulars which Dr. Mercier has detailed should be especially observed by the druggist who sends the article, and the person who collects it; by attending to the distinctions described in this memoir, the clean, pure, flowers may be readily known. Though the arnica montana has no place in the London Pharmacopœia, it is admitted into those of Edinburgh and Dublin; is much used on the continent, and certainly possesses very active powers, which might be applied to good purpose in the cure of many diseases.

---

*Observations on the curative and anti-contagious Properties of oxygenated Muriatic Acid. By* M. GUYTON MORVEAU.

This enlightened chemist commences his inquiry with quoting a large portion of Mr. Braithwaite's Observations on the Utility of the oxygenated Muriatic Acid in the Cure of Scarlet Fever, inserted in the 18th volume of the Philosophical Magazine. Mr. Braithwaite there observes, "Since the use of this medicine I have never had recourse to emetics, purgatives, blisters, or diaphoretics; a regular perseverance in the oxygenant remedy has universally succeeded, my patients rapidly recovering, and being seldom afflicted with those complaints succeeding the scarlet fever, such as pains of the joints, paucity of urine, and universal anasarcous swellings."

M. Estrilaud, M.D. of the university of Montpellier, employed the oxygenated muriatic acid with signal success in the treatment of four thousand Spanish prisoners, affected with fevers of the worst species and most malignant character, at Carcasonne.

In an account of it which he transmitted to the war minister, he states, that he was induced to have recourse to this remedy, after having experienced the inadequacy of the usual modes of treatment, and being convinced by observation, that the fumigations which he daily found salutary as preservatives, could not act constantly, nor with sufficient intensity, upon the contagious miasmata continually reproduced in abundance, in places crowded with infected patients. His treatment chiefly consisted in the internal use of the oxygenated muriatic acid, in the dose from six to eight drams, in a pint * of mucilaginous decoction. From this he affirms he has never seen any ill effects result; on the contrary, the most alarming symptoms have disappeared, and the patients have advanced rapidly in their convalescence, with a keen appetite; in short, he ranks the efficacy of this remedy in malignant fevers, with that of cinchona in intermittents.

The author next considers the action of oxygenated muriatic acid upon hydrophobic virus; but does not adduce a single fact from his own experience in its favor. As an external application, it may act like caustic, and prove useful by destroying the affected parts. M. Cluzel, indeed, has asserted, that some persons in the hospital at Bourdeaux, bitten by a mad wolf, were saved by taking the remedy internally, but not the slightest evidence appears of either themselves or the animal being affected with hydrophobia.

M. Morveau concludes with some cases of psora cured by the application of this acid. In a note, he slily insinuates his surprise, that, in all the English publications that have

* We suspect a quart is the measure; as the author says "*pinte (litre)*", and we understand that a *litre* is about two English winepints.

 lately lately appeared, in which the means of destroying contagious miasmata are considered, fumigations with the oxygenated muriatic acid, are only indicated; whilst those with nitric acid are not mentioned; and that Mr. Parke, in the fourth edition of his Chemical Catechism, published at London in 1810, appears only to place confidence in a preservative apparatus for disinfecting the air, which he describes under the name of *Morveau's preservative phials!*

---

*Journal Général de Médecine, de Chirurgie, de Pharmacie, &c.*

Avril et Mai, 1811.

(Continued from Vol. 27, p. 159.)

Dr. Carron relates some cases of periodic head-ach, which were cured by bark and opium. In one case, where bark did not agree with the stomach, arseniate of potass, or Fowler's solution, was given in the dose of twelve drops every six hours during the interval of ease. The first paroxysm was considerably diminished; the second was very slight and terminated the complaint.

M. Marcescheau has detailed the particulars of the case in which an hæmorrhoidal affection, accompanied by hæmoptoe, was cured by the establishment of the hæmorrhoidal flux, excited by art.

The patient, Miss Desoras, by profession a painter, was subject to a periodical hæmorrhoidal discharge, which governed her health, although the catamenia were regular. She was of a lively disposition, warm imagination, and loved her art with enthusiasm; sacrificing to its pursuits her hours of repast, of sleep, and of exercise.

After a continued series of excessive application, in January 1806, she was seized with violent head-ach, swelling about the eyes, and slight deafness: she also felt pains in her loins; her pulse was hard and contracted; the urine red and turbid; stools hard and infrequent. A medical practitioner, being called in, bled her from the arm. She was relieved for a few hours, after which the head-ach returned with great violence, accompanied with fever, delirium, and oppression. Her feet were now placed in water; glysters were thrown up, and she experienced momentary relief. Apozems, in which was cinchona, were prescribed; she took them with extreme repugnance; they occasioned a great inclination to vomit; a blister was applied to the arm, but produced no other effect than great pain. The catamenia, for the first time, were suppressed. A few drops of blood passed from the nose, and she also spit blood. She rapidly grew emaciated, her strength diminished, and she was obliged to to continue in bed; palpitation of the heart occurred frequently; the general oppression became more urgent, and the spitting of blood more considerable.

So rapid was the progress of this complaint, that, when M. Marcescheau saw her on the 2d of March, six weeks after her attack, he found the lightest drink could not be retained; the pulse was scarcely perceptible; the voice lost; the breath, even at some distance, had an insupportable fœtor; the whole body was in a state of marasmus, and the hippocratic countenance announced approaching dissolution.

Madame Desoras, the aunt of the young lady, and the attendants, considered her to labor under pulmonary consumption. It was proposed to apply a blister to the chest. M. Marcescheau had remarked, that the blood expectorated was black and frothy, and mixed with mucus. Upon inquiry if, during the course of her illness, any pus, grumous matter, or concretions, had been spit up, he was informed that the expectoration for four or five weeks since the patient spit blood, was as he then saw it.

It appeared to him that the affection of the lungs, and the other symptoms, were owing to a sanguineous hæmorrhoidal metastasis. Though there were no external hæmorrhoids, nor any discharge, the pains in the rectum and in the loins, which Mlle. Desoras had experienced from the beginning of her illness, were sufficiently indicative of internal hæmorrhoids.

From the extreme weak state of the patient, M. Marcescheau only ordered a single leech to be applied to the rectum; and the blister on the arm to be discontinued. He directed her diet to consist of barley-water, milk-whey, and chicken broth.

The blood drawn by the leech relieved the oppression, and the pulse slightly rose. In the evening three more leeches were applied, and procured a copious evacuation; hæmorrhoids formed externally, and the patient experienced acute pains in the fundament. In a few days she recovered some degree of strength, the stomach resumed its functions, and she began to eat soup, vegetables, and at length roast and boiled meat. In the space of three months and a half leeches were applied to the fundament seventeen times, amounting in the whole to one hundred and three, when the hæmorrhoidal discharge appeared for the first time, and was pretty abundant. The catamenia became regular, and the patient regained her health, strength, and *embonpoint*. Returning with eagerness to her former pursuits, she continued sitting before her models for six or eight hours, with her pencils in her hand. In July 1806, she had an hæmoptoe, and no hæmorrhoids.

morrhoids. Two applications of the leeches brought back the hæmorrhoidal discharge, and the hæmoptoe ceased.

The same cause continuing to operate, in October 1805, she spit blood in an alarming manner, notwithstanding the repeated application of leeches. Her aunt, Mad. Désoras, believed that the hæmorrhage proceeded from vessels ruptured in the chest. M. Dubois, professor in the faculty of medicine, was called in. He agreed with M. Marcescheau respecting the cause of the malady, and proposed, with the view of recalling the hæmorrhoidal discharge, irritating glysters with common salt. They had the desired effect; the flux returned, and the spitting of blood diminished, but did not entirely cease.

At this period a regular tertian intermittent supervened, the spitting of blood returned, and the patient became extremely weak. Bark was given in spite of her aversion to it, and the fever soon yielded to the remedy; but the spitting of blood not having in the least abated, leeches were again applied to the fundament. The hæmorrhoidal discharge was effected a second time, and has since recurred periodically every six weeks, without altering the usual menstrual evacuation. Whenever the hæmorrhoidal flux was retarded or diminished, the patient spit blood, experienced great inconvenience, and, to prevent worse consequences, was obliged to submit to the application of leeches to the fundament.

In April 1810, Mlle. Desoras, having applied with more than ordinary diligence to her professional pursuits, continuing at work for twelve or fourteen hours a-day, was affected with a total suppression of the periodical hæmorrhoidal flux, which besides the usual symptoms now occasioned a new one, and was complicated with a quartan fever. The additional symptom began with great paleness and anxiety, which ended in syncope, which continued two or three minutes, and sometimes longer. As soon as she regained her senses, she felt perfectly well sometimes for several hours together, at others only for a few moments, when the syncope recurred.

This symptom was followed by an intermittent fever, which continued at the time she was affected with the quartan, though it had no correspondence with the paroxysms of the latter. Considering it as a nervous affection, M. Marcescheau ordered her to use the warm bath, which, however, made her decidedly worse, and she was considered in danger. The quartan fever acted with increased violence, and she now requested to take the bark, which soon terminated the complaint; complaint; and the hæmorrhoidal flux was again restored by the means formerly employed.

Thus two different diseases co-existing were treated, at the same time, as if they had occurred in different subjects.

From the scanty information in the two last numbers which we have been able to obtain of this long-established Journal, and from the meagre announce of new medical publications in the report of the Imperial Institute for 1811, we conclude that medical literature is not in a more flourishing state on the continent than it is in this country.

---

*Observations on the contracted Intestinum Rectum, and the Mode of Treatment, accompanied with Cases illustrative of the different morbid Appearances attendant on the Complaint. To which are subjoined, two Engravings of the Disease. By* W. White, Member of the College of Surgeons, London, and one of the Surgeons to the City Infirmary and Dispensary, Bath. Small 8vo. Bath. 1812. pp. 86. two plates.

This little practical work is divided into seven sections. The first section consists of general remarks on the contracted rectum. Dr. Sherwen, Mr. White considers as the first person who wrote a history of the contracted rectum; and his paper on the subject was printed in the second volume of the Memoirs of the London Medical Society. It does not appear, however, that Dr. Sherwen was acquainted with the disease in its early stage, or under the form of simple stricture. But, as it is evident that some of the old practical writers were acquainted with this disease, and that Mr. Pott, in his lecture on diseases of the anus, notices stricture of the rectum, Mr. White can mean only that Dr. Sherwen was the first who wrote expressly on the subject. Since the period of Dr. Sherwen's publication, the disease has been frequently noticed; and the profession is particularly indebted to Mr. Copeland for a more correct knowledge of "stricture of the rectum," as elucidated in his "Observations on the principal Diseases of the Rectum and Anus," published in 1810. According to Mr. White, the contracted rectum is a rare disease, for, out of 37,000 patients who came under his immediate notice in the course of twenty-four years, only fourteen cases of this disease occurred, or something more than one in two thousand. But Mr. White does not intend to acknowledge or admit its unfrequency, but rather that it has been misunderstood and overlooked. In this stricture-making age, however, the probability is that stricture of the rectum has often been imagined, and that the ruling

 passion passion has often created when it intended to describe a natural fact. Be this as it may, the disease occurs often enough, and is of sufficient importance to interest the public in all investigations concerning it.

The second section contains "Remarks on the Diagnosis of the Disease." The insidious nature and slow progress, the similarity of symptoms arising from other causes affecting the intestinal canal, render the pathognomonic signs of the disease so precarious and uncertain, that we apprehend Mr. White has much benefited the profession by giving the symptoms as they arise.

Those which more particularly indicate the presence of this disease in its early stage are, says Mr. W.

"Habitual costiveness, occasional uneasiness arising from a sense of fullness in the course of the transverse arch of the colon, but more especially towards the termination of the sigmoid flexure; the patient is also sometimes sensible of the aggravation of this symptom, from the quality or quantity of the food which is taken. There is also an uneasiness in the rectum on going to stool, attended with some difficulty in voiding the fœces. As the disease advances, the alvine excretions become gradually more scanty; the fœces are smaller figured than those which are natural, and are often discharged with a squirt. After an evacuation, a sensation commonly continues for some time, as if the whole of the fœces had not been expelled, which by degrees goes off, until the next time of going to stool, when a similar sensation occurs."

To these symptoms we shall add a train of phenomena characteristic of this disease, as given by Dr. Robert White in the fourth volume of the Memoirs of the London Medical Society.

"When a person somewhat advanced in life, is troubled with frequent constipation, complains of fullness and weight in the stomach, with repeated inclination to discharge the contents, and uneasy rumbling in the belly and distention in the lower part of it, with a sensation of numbness toward the upper part of the sacrum, extending down the rectum; repeated fruitless efforts being also made to pass a stool, attended with a sense of constriction and tenesmus high up in the rectum, and flatus, which seemed to the patient to occupy the intermediate space, bursts forth; clysters failing as well as medicines, and the complaint unattended with fever or pain; it will be reasonable to expect some mechanical obstruction in the passage."

Dr. Sherwen, in the publication before cited, considers diarrhœa as one of the earliest symptoms of this disease: this, however, Mr. White controverts, and observes that the disease will be found to be in a very advanced stage, whenever a spontaneous diarrhœa takes place. We would particularly cularly press on the attention of our readers the diagnosis of this disorder, because it frequently exists for a long period before it is detected, and acquires a permanence and stability to resist the curative process, which, perhaps, do not appertain to its early stage. It must be distinguished from the schirro-contracted rectum, from schirrus uterus, from diseased prostate, and from dysentery. Dr. Sherwen and Dr. Robert White, in the Memoirs of the London Medical Society, Siebold Dissertatio de Morbis Intestini Recti, Wrisberg de preternaturali Intestini Recti, &c. Mr. Copeland on the Diseases of the Rectum and Anus, with the work now before us, will afford the means of understanding this disease, as far as printed details go: the book of nature must supply the rest.

The third section treats "*of the Modes of Examination, and the usual morbid Appearances.*"

The actual state of the intestine is only to be ascertained by the *tactus.* Whenever we have a suspicion of the complaint, a manual investigation must be employed.

"This ought to be performed in the most careful and attentive manner, seeing there is a possibility of mistaking the complaint either for a diseased prostate gland or a schirrus uterus, especially if the hardness and tumefaction is attached to the cervix uteri or back part of the vagina. In prosecuting the examination, the first step to be taken is to introduce the finger (oiled) as high up the rectum as possible, at the same time desiring the patient to bear down, as if going to stool. For, if the examination is first made by introducing a bougie, it may happen, that the instrument is pushed between the folds of the intestine, particularly if there should be particular laxity of its internal membrane; and the practitioner may be led to suppose there is a stricture, when in reality none exists. If, however, on introducing the finger, neither stricture nor induration can be discovered in the rectum, a small-sized bougie must be introduced, and passed as high as the termination of the colon; because there may be a stricture at that part only, although we commonly meet with one, two, or three, inches lower. And this I believe will generally be the case, when the superior stricture has been of long standing: analogous to what happens in strictures of the urethra.

"Sometimes it happens that the gut is so much contracted as to render the introduction of the finger impracticable, and the passage will only admit a middle-sized urethra bougie, and sometimes only a small probe."

The diminution of the calibre of the rectum is produced by various alterations in its organisation. Sometimes there is only a diminution of the diameter of the canal, perhaps, in its whole length; at others, annular strictures are discovered, or schirrus surrounds and fills up its cavity; and sometimes tubercles obstruct the passage. For further observations, servations, and the morbid appearances detected by dissection, we must refer to the pamphlet.

The fourth section contains the Prognosis. When the stricture is simple, does not extend beyond the sigmoid flexure of the colon, and the patient's general health is unimpaired, the prognosis is favorable. If the disorder is of long standing, the intestine much thickened and indurated, and the general health sensibly declining, with sallow countenance, hectic pulse, &c. the prognosis is unfavorable. Mr. White speaks positively here to an important fact; *whether the disease be simple stricture or confirmed schirrus, it is equally fatal if left to itself.* This fact is confirmed by Mr. Copeland.

Section five inquires into the "Cause of the Disease."

Mr. White asserts, that the complaint is produced "by a morbid action essentially different from that of inflammation. It appears (he adds) highly probable that the glandular structure of the rectum may form the predisposing cause of the disease; and it is also presumable, that the accumulation of hardened fœces, and the pressure occasioned by their passage through the intestine, and the violent straining thereby induced, may prove the general exciting cause."

The method of treatment is explained in the sixth section. As it is evident that a dilatation of the contracted passage is the *sine qua non* of the cure, a strong analogy has led to the mechanical means employed in other strictured parts; but these require to be used with discrimination, caution, and considerable reserve. The author examines with freedom the methods employed in this case by others; and the opinions and practice of Wiseman, Pearson, Desault, Sherwen, Rob. White, Darwin, Charles Bell, and Copeland, pass in review.*

The treatment of the disease, as laid down by Mr. White, is divided into two branches, general and medical, and local or chirurgical. The great object in both is to keep down pain and irritation while the distensive means are carrying on, or the remedies employing to remove the disease if it have a specific origin. Bougies, &c. are only admissible when they can be employed without creating great pain and

---

* We are concerned to observe that Mr. White thinks Mr. Copeland has not done him strict justice; but our knowledge of that gentleman enables us to say that we believe he did not intend to excite any feeling of that kind, but to make his observations with perfect candour. If the contrary appears, it must have arisen from some unintentional turn of expression, too frequently the result of the imperfection of language, inadequate to give a precise and faithful portrait of thought.

irritation; irritation; sometimes the stricture, if not longitudinal, may be divided by the knife; emollient enemas are always useful; and, where anodines are required, the *extractum Hyoscicami* is to be preferred, as it has not the property of occasioning constipation.

The seventh section is particularly valuable by its facts, containing fourteen cases of contracted rectum, treated by the author; to which two plates are annexed, shewing the appearance of the diseased intestine as ascertained by dissection.

---

*An Essay on the Probability of Sensation in Vegetables. By* J. P. TUPPER, F.L.S. and Member of the Royal College of Surgeons. 8vo. Lond. 1812. pp. 142.

This work embraces a variety of subjects, on which the author has offered such observations, as evidently shew that he is capable of thinking for himself. The language is simple, elegant, perspicuous; free from technical obscurity, and affected embellishment.

Mr. Tupper begins his Essay with the following introductory observations, to which he has affixed a very appropriate motto from Armstrong.

> " ———————— in a doubtful theme
> Engaged, I wander through mysterious ways."

" It is as difficult to ascertain the nature of vegetable existence, as to determine what constitutes the living principle of animals. It is evident, however, that life is intimately connected with a particular organic structure of parts; for through the medium of that organisation existence itself is preserved.

" The physiologist, who investigates the laws which regulate and direct all the different movements of the animal machine, cannot observe without admiration its wonderful fabric, which, from a mere *rudis indigestaque moles,* the secret working hand of Nature has elaborated into so complicate a form, every part of which is most exquisitely finished, and the whole so well and skilfully arranged, as to constitute a being capable of giving existence to others similar to itself.

" Although the vegetable physiologist may not have more to engage his attention, yet he has not less to admire. How widely and wonderfully different is the mature vegetable from the seed which gave it being! How great the contrast between the diminutive acorn and the stately forest oak! The seed is seemingly nothing more than a mere homogeneous substance; but, when placed within the influence and operation of particular causes, its latent vital principle is called forth into action, a variety of organs are unfolded, and by successive evolutions the plant arrives at that state which constitutes the perfection of its nature, when, like animals, it is also endued with the power of propagating its species."

The author then proceeds to consider the distinction between animals and vegetables, gives a general view of their analogies, and points out many interesting particulars. Observations on vegetable motion, and on instinct and volition succeed; in the course of which Mr. Tupper manifests many original and interesting ideas. The other subjects treated of are arranged in the following order: Of vegetable instinct; of the sleep of plants; of sleep in general; of sleep as relating to voluntary power; of locomotive power; of sensation in general; of vegetable irritability; of the nervous system of vegetables; of vegetable sensation; objections considered; the exposure of animals to injuries; of the limits prescribed by nature to the destruction of life; of vegetable self-preservation; organs of defence in vegetables; effluvia of plants a protection from external injuries; of the preservation of animal and vegetable life; of the limits between animal and vegetable creation; conclusion.

The author has also enlarged on some of the above subjects under the head of "additional observations," which are chiefly of a metaphysical nature, and display much ingenuity of argument, although some of the opinions advanced are disputable. On the subject of instinct, he observes,

"From this view we may form some idea how far instincts may supply the deficiency of intellectual power, and even compensate for the total want of reason in the brute creation. But where will be found any power or quality, as a substitute for sensation? The idea of instinct is naturally associated with that of life, and the idea of both, either jointly or separately, with that of *sensation:* and, as sensation does exist in animals independently of those eminent attributes with which it is combined in our natures as rational agents, may we not reasonably infer that vegetables have likewise their share of sensitive power, and consequently the means of enjoying their existence?"

Our limits will not allow us, at present, to make further extracts from this ingenious publication; but we refer our readers to the work itself, for many interesting remarks and curious facts.

---

### *Edinburgh Medical and Surgical Journal, No.* XXIX.

(Continued from p. 334.)

*Case of Torpor, in which Dissection did not discover any satisfactory Explanation of the Symptoms. By* W. Cooke, Surgeon, Plaistow.—The patient, a woman 49 years of age, whose case is here detailed, was a sufferer for seven or eight years, under circumstances that indicated affection of the head. From September 1809, to September 1811, she had been confined, more or less, with attacks of a disease approaching proaching to apoplexy, accompanied with extraordinary torpor. On the 6th of the last-mentioned period she expired. The *sectio cadaveris*, on the 9th, exhibited the following appearances.

"The body was considerably changed.

"The brain was soft, but not so much so as to prevent examination.

"There was no morbid appearance on the dura mater. The vessels of the pia mater were rather turgid, and, at the anterior and under part of the right hemisphere, the turgescence was by far the greatest.

"*There was no unnatural effusion.*

"The ventricles contained a very little more fluid than usual; the posterior horn seemed rather enlarged.

"When the brain was inverted, we found about an ounce of aqueous fluid collected in the basis cranii. The tunica arachnoides between the cerebellum was considerably thickened, and the vessels of the pia mater in this part more turgid. There was also one small clot of blood, perhaps the size of a large pea.

"There was no alteration in the structure of the brain.

"The abdomen had become greatly distended in the course of the last 24 hours from beginning decomposition; but we found all the viscera in a perfectly natural state.

"The stomach, intestines, and liver, were particularly examined, and appeared decidedly healthy.

"The gall-bladder was distended with healthy bile. Perhaps there is no impropriety in mentioning, that the appendages to the uterus on the left side were of a dark red color, but on the right they appeared as usual."

---

*Case of Inguinal Aneurism cured by tying the external Iliac Artery. By* W. Goodlad, Surgeon.—This case gives another instance of the success of this bold, and once thought impracticable, operation, performed, not in the metropolis, but in a remote province; indicating how much surgical science is spreading throughout this island. Mr. Goodlad thus describes the steps of the operation:

"An incision, upwards of three inches in length, was made through the integuments, beginning at the upper margin of the tumor, about three inches from the symphysis of the pubes, and carried almost directly upwards. In the dissection of the cellular membrane, covering the aponeurosis of the external oblique muscle, several arteries were divided, which supply the adjacent glands. These vessels were immediately secured, and more than two inches of the tendinous expansion exposed. Its fibres were carefully divided and dilated downwards, by the probe-pointed bistory introduced upon the finger. The artery was now clearly distinguished by its pulsation. The fibres of the external oblique, with the margin

 of of the internal and the transversalis muscles, were also divided upwards, to allow sufficient space for the two fore-fingers of the left hand to be placed in contact with the arterial sheath.

"I endeavored to detach the fascia, so as to be enabled to pass my finger round the artery. In this attempt considerable difficulty was experienced; but, by keeping the artery firmly in its situation upon the psoas muscle, with my finger and thumb in contact with it, I succeeded in passing the eyed end of a probe (about half an inch of which was bent to a right angle) under the artery, from within outwards. The shaft of the probe was gradually bent likewise, to facilitate its turning in the wound. A double ligature being put through the eye, the probe was redrawn. The upper ligature was tied as high as possible by Mr. Killer. The pulsation in the tumor immediately ceased. The lower ligature was also tied, but, as the space between the ligatures did not appear to warrant the divisions of the vessel, it was left entire. The integuments brought into contact were secured by a couple of stitches, stripes of adhesive plaister were applied, and a roller passed round the whole. The patient was put to bed with the thigh bent upon the pelvis. The temperature of the limb had been kept up during the operation, by a flannel roller applied round it."

At the end of three weeks the wound was little larger than a pea; the discharge was trifling. At the end of a month the aneurismal tumor had decreased one-third; the wound was perfectly healed; the patient gaining strength, and able to walk two miles in a-day. It may be right to state that two surgeons had been consulted prior to Mr. Goodlad's seeing the patient, one of whom advised removal of the tumor by extirpation, the other the frequent application of blisters to it.

---

*Case of Erythema Mercuriale, accompanied by an Affection of the Cornea. By* J. Nicholson, Surgeon.—The patient, a private in the Derbyshire Militia, had chancres and phymosis, June the 6th. On the 8th gonorrhœa appeared. On the 18th the penis became painful, swollen, and inflamed. On the 22d the tumefaction and inflammation of the penis continued. The lower part of the abdomen, the groins, and upper part of the thighs, were observed to be covered with innumerable papulæ of a bright red color, accompanied by a very distressing itching sensation. The tongue white; bowels costive; pulse 120, quick and full; skin hot and dry; and great thirst. On the 29th the papulæ were extending over a larger surface. Numerous small vesicles, containing a serous fluid, appear on the thighs and lower part of the abdomen. 26th. The erythema became universal; the palpebræ and tarsi swollen and inflamed. 28th. A slight exudation from the groins and scrotum yesterday, which is

 now now increased in quantity, and become ichorous. The eruptive disease proceeded through, as it should seem, regular stages; and on the 6th of July, about fourteen days from its commencement, the cuticle was entirely desquamated. On the 4th of July, the cornea had nearly lost its transparency, appeared dry, and exactly resembled an eye which had been exposed to the air for some time after death. By the use of antiphlogistic remedies at first, and cinchona at the subsequent period of the disease, the patient perfectly recovered from the erythema; and the corneæ were also restored to a perfect transparency.

---

*Case of Chorea cured by Purgative Medicines. By* H. SHUTE, M.D. Bath.—It is only necessary to announce this as a useful fact, in the history of an obstinate disease.

---

*Observations upon (the) Venereal Disease. By* J. PEAKE, Surgeon.—These observations are intended to prove that an absorption of the syphilitic virus may take place, producing bubo and even ulceration of the throat, without any antecedent local affection.

---

*Case in which the beneficial Effects of Friction, as recommended by Mr. Grosvenor, of Oxford, were strongly exemplified. Communicated by* Dr. DUNCAN, senior.—This case is related by the mother of the patient; and, as it gives a distinct and unaffected history, we shall present it to our readers in the lady's own words.

"Your little patient, Margaret, got a bad knee, after the scarlatina she had, when in Albany-street. I believe the last time I wrote to you, was to take your advice for her, in 1807, from St. John's in Yorkshire. During our stay in that country, Margaret got infinitely worse; the knee swelled to a great size; the lower part of the limb became proportionably wasted; her health gave way; general debility, loss of appetite, quick pulse, morning perspirations, and heightened complexion, seemed to portend the most unhappy termination.

"Mr. Hey, of Leeds, a surgeon of eminence, visited her, and seemed to think very badly of the case. He recommended an immediate removal to the sea-shore, and to a warmer situation. We removed to Parkgate, in Cheshire, in May 1808; and Margaret received considerable benefit from sea air, bathing, and exercise. Her general health seemed established, and we remained there with her till late in the month of October. That winter passed tolerably with her, in Chester, but in the spring she got bad again. We brought her to Buxton in March, and from that to Parkgate in June 1809; the air and water seemed to have the same beneficial effect effect on her general health, but she was not better of her lameness; on the contrary, the leg was so contracted, so incapable of bearing any part of the weight of her body, or indeed of touching the ground, that she was obliged to use a crutch; her knee was frequently bled with leeches, and tepid water poured over it, according to the directions of Dr. Monro, while calomel, hemlock, lime, &c. were tried without any success. Still, however, there was *no sore;* and, assured of the consequences which might arise from *the skin being broken,* I was particularly careful never to let any remedy be used likely to produce that effect; of course there was neither blisters, caustics, nor issues. Appearances, however, indicated that sores could not long be prevented; the knee got very much discolored, and *pointed* in several places, underneath and among the tendons on one side.

"Under these circumstances she was seen by the elder Dr. Currie, of this city, who mentioned to me Mr. John Grosvenor, a surgeon of eminence in Oxford, as a person whom he knew to have performed some remarkable cures. I wrote to him stating Margaret's situation, and, though he gave me little or indeed no encouragement, (for he said it was in incipient cases his plan was mostly beneficial,) I brought her to Oxford, December 1809. After staying with her a few weeks, and Mr. Grosvenor's telling me '*he had cured as bad,*' I took a lodging for her, and left her with a confidential servant under Mr. Grosvenor's care.

"The result was, that she escaped the usual spring attack, and at the end of seven months, in July following, returned to me, walking without either crutch or stick, laying her heel flat to the ground, and with but little lameness, which a constant perseverance since in his mode of treatment has *entirely* removed. She is at this moment perfectly free from lameness, or disease of any kind; that leg as large as the other, and little or no difference in the figure of the knee.

"Friction, exercise on foot, and the occasional use of calomel, were the only remedies used, and are to be continued till she attains her full growth. She is now in her 13th year. I know, my dear doctor, from my former experience, that you will sympathise with Mr. W. and me in the happy termination of a disease which I began to consider as incurable."

---

*Observations on the Occurrence of Small-pox after Cow-pox. By* T. C. Haden, Surgeon.—These observations go to establish the fact that the *Vaccina,* if it does not, in every instance, prevent the accession of variola, always modifies that disease, and lowers its virulence. The writer of the paper thinks he has proved by his cases,

"1st, That the cow-pox is capable, not only of destroying, in most cases, the pre-disposition in the human constitution to be affected with small-pox, but also, in others, to modify the appearances of of small-pox, to shorten its duration, and almost to change it into a new disease.

"2d, That, although this modification of small-pox is mild in itself, yet it is capable of producing, in others predisposed to the disease, a small-pox similar to the common kind in its symptoms, its duration, and in its terminations. Hence they are one and the same disease.

"3d, That the modified small-pox is subject in its duration to a law as certain as that which regulates the termination of common small-pox."

---

*Case of Hepatitis terminating in Sphacelus. By* W. C. Surgeon.—To a narrative authenticated only by initials, we can give but little attention.

---

*An Account of the Proceedings for establishing Sea-water and other Baths, and an Infirmary in the Vicinity of London; with the Prospectus, Engineer's Report, and Medical Certificates, &c. &c.* Lond. 1812.

Although not strictly medical, we thought that a short notice of this pamphlet might be interesting to some of our readers. From it we learn, that, sixty years ago, a proposal was made to supply London with sea-water from the coast; that since that period the project has at different times been thought of; and that last year it was seriously resumed. A party was then formed to carry the measure into effect; and, from the engineer's report, it clearly appears that the scheme is practicable. The site of the proposed baths is Copenhagen-fields. The distance from which to the coast of Essex, on the line intended for laying the pipes, &c. is 37 m. 3 f. and the water is intended to be carried through this space as under:

"From Shoeburyness to Leigh, 3 m. 2 f. 32 p. through a brick tunnel two feet diameter, passing under the sand, on the beach, so as in no wise to interrupt the approach of barges, &c. or to be injured by them. There will be proper means provided for cleansing this tunnel.

"From Leigh to Vange, 8 m. 5 f. 28 p. it will pass through an open cut four feet wide and three feet deep.

"From Vange to the reservoir on Langdon-hill, 2 m. 5 f. 24 p. it will be forced by an engine, through a line of pipes twelve inches diameter, properly adapted to the pressure to be sustained.

"From Langdon-hill to the ultimate reservoir at Copenhagen-house, 23 m. 2 f.; it will descend through a line of pipes nine inches diameter, properly laid and secured at the joints, in the most substantial and effectual manner; and, in order to ascertain that the pipes are perfectly sound and good, they will be proved, previously to and after they are laid down, by applying a force at least double that that which will afterwards act on them; and, notwithstanding which, valves will be so applied as to prevent the possibility of any very considerable quantity of water from escaping, even should the pipes burst; which is extremely improbable, after the above precautions. In answer to those who doubt the stability of the summit reservoir, it may be observed, that the East London water-works, and Tottenham-court-road reservoirs, are merely *artificial mounds*, the base of them probably not exceeding two acres; while Langdon-hill is a substantial work of nature, existing from time immemorial, and of many thousand times that area.

"2nd. In order that the water may flow into these baths as pure and pellucid as possible, it is intended that several reservoirs shall be formed through which it will pass in succession, remaining a considerable time in each, that any extraneous matter it may contain may be deposited or otherwise separated, before the water is raised into the summit reservoir, during which, evaporation will tend to encrease, rather than diminish, the saline properties of the water. And that it may enter the exterior reservoir in its pure saline state, it is intended that none shall be admitted until the tide has flowed up four hours, by which means it will possess its full portion of marine properties; after having passed through these several reservoirs, it will be raised to the summit reservoir, on Langdon-hill, (which is considerably higher than Copenhagen-fields,) and from thence it will descend by its natural gravity into the ultimate reservoir, for the immediately supply of the baths.

"3rd. That no interruption may occur by the accumulation of air in the pipes, valves, on a peculiar construction, will be applied to every necessary point, as well as other valves and outlets, for the purpose of cleansing, &c.; and the following deductions will shew that the quantity of water delivered in a given time will not be diminished by the increased length of the pipes, while their inclination to the horizon remains the same."

Several certificates from eminent medical practitioners are adduced in favor of the plan: from these it results,

"That the formation of sea-water and other baths, in the manner proposed, would provide the physician and surgeon with additional means of extending the benefit of their art to a large and afflicted class of people of every rank; that they would impart vigour to the constitution impaired by dissipation, enfeebled by hereditary disease, or suffering from the effects of repeated indisposition; whilst by promoting gentle and agreeable exercise, cleanliness, and hilarity, they would powerfully contribute to preserve the health of those who already enjoy it; for, as Clemens Alexandrinus has well observed, there are four reasons why every person should use the baths,

ἢ καθαρότητος ἕνεκα, ἢ ἀλέας, ἢ ὑγείας, ἢ τὸ τελευταῖον ἡδονῆς

for the sake of cleanliness, of warmth, of health, and, lastly, of pleasure.

"The claims of the infirmary on public sanction are obvious and paramount.

paramount. Independently of the relief it would afford to the numerous lame and diseased objects, recommended by its own governors, it might constitute a sort of hospital of ease, to those great and liberally endowed institutions in the metropolis, which almost constantly have several patients lingering in their wards, whom sea-bathing and change of air* would either altogether cure, or at least restore to a degree of strength, which would enable them to return to their families and occupation, while their beds in the hospital would, in the mean time, have been occupied by a succession of objects whose cases admitted of more speedy relief."

Notwithstanding the important benefits which would thus accrue to the inhabitants of London, notwithstanding that not the shadow of an argument was opposed to the measure in the House of Commons, the bill was thrown out by a majority of twelve. But we learn that the gentlemen engaged in the undertaking, are still confident of ultimately succeeding, and intend to persevere in a measure which has obtained very high sanction, and certainly deserves encouragement.

---

*A Series of original Experiments on the Foot of the living Horse, exhibiting the Changes produced by Shoeing, and the Causes of the apparent Mystery of this Art. By* BRACY CLARKE, Veterinary Surgeon, F. L. S. In Two Parts. Part I. 4to. Lond. 1810.

Mr. Clarke, well known for his attention to natural history, as connected, particularly, with the horse, and to the anatomy and diseases of that animal, purposes to give a series of dissertations on the alterations of structure, and on the complaints of that animal, resulting principally from his artificial state, as under the controul of man. The treatise before us is one of those dissertations, and on a subject of great importance. In order to have a point of comparison from nature, Mr. Clarke's first object has been to ascertain the exact form of the foot of this animal when unchanged by art. With this view he took casts in plaster, from the healthy foot of a young mare, permitted to run unshod to her fifth year, and by repeating those casts from one period to another, whilst the foot was under the influence of the shoeing process, he became accurately acquainted with the changes that took place, and the degree of diminution of the foot in a given time. The figures of the foot thus obtained, are represented in four excellent plates. On comparing the

* It must be considered, that the site of the proposed infirmary will be on an elevated situation, deemed, by the best-informed persons, the most salubrious in the neighbourhood of London, from which it will be at an advantageous distance.

condition of the foot in its natural state, with its condition a year after the first application of the shoe, the following alterations were found to have been produced.

"The elastic parts of the heels have lost their swelling, rounded, and beautiful, appearance, by the sinking of the cartilages, and the loss of the peculiar elastic matter within; and the surface they now present is an ugly flat slope towards the base of the frog. The horny heels from one to the other, in the original state of the part, measured somewhat more than four inches; in the second cast scarcely three. The foot, measured across in its widest part, viz. at the greatest swell of the quarters, was in the original cast nearly five inches and a half; in the second cast it was four inches and seven-eighths. The actual length of the foot, we may remark, has not been much changed; which seems to confirm the circumstance that the cause operating these effects has been lateral principally, and seems to evince its having been the effect of nails. The frog had lost, through its being wasted and cut away by the smiths, the rounded swelling and projection we have distinguished by the name of cushion; and its lower surface, though its substance was so much diminished, was lower by near one-fourth of an inch, than the horny heels or swell. This appeared to arise in part from the condensation of the horn of the heels, from the constant pressure of the shoe upon them, and also from the circumstance before on the cause of the descent of the frog. The texture of the frog, from an agreeable yielding elasticity, had become hard and unyielding to any impression of the fingers; and its sides, which at first were gently inclining or sloping to the commissure, had become almost perpendicular. The cleft at the base of the frog had become partly closed, forming a rounded ill-formed hole, and much deeper than the cleft of the natural foot. The base of the frog, which was, in the natural foot, of the width of two inches and a half, had now become hardly as much as two inches. The bars had considerably lost their sloping direction, and had become more perpendicular, and encroaching upon the sides of the frog, and consequently more disposed to compress it. The sole appeared somewhat more arched or cupped than formerly, but the degree of expansion it had undergone, as also the elasticity it had lost, could not be accurately ascertained in the living subject. Thus we see the beautiful and useful symmetry of Nature's mould, no part of which is without its use, has been changed by artificial restraint to deformity and incompetence."

The mode of operation by which shoeing produces the changes above stated, Mr. Clarke details with a precision suited to the accuracy of his observations of the progress of the alteration. The first and most obvious evil he considers to be the permanent application and constant pressure of the inelastic shoe against the bottom of the foot. The nails in the sides, by being immoveably blocked in the perforation of the shoe, is the next evil. They create a solid resistance of of iron in this part, and prevent the natural expansion of the hoof; and they nearly destroy every movement of the posterior parts and heels, by keeping the quarters fixed. Our author attributes the greater part of the mischief done the foot to the shoe and nails, and allows very little to standing in the stable. Though we may not be able to subdue an evil, it is certainly desirable to know its exact nature and progress; and, if we do not see, at present, how the iron shoe and nails can be rejected in this country of artificial roads, we may trust to our ingenious author for some modification of the method of shoeing, that may postpone the evil. But for a knowledge of this, we must wait the progress of Mr. Clarke's design.

As a writer on subjects connected with his particular destination, we have long observed Mr. Clarke with attention and satisfaction. His close observance of nature, and his development of obscure facts, have acquired for him a reputation, which his present production has not diminished. We remember his paper on the genus *œstrus*, in the Linnæan Transactions, with peculiar pleasure. The following remark contains a fact of much interest, and with it we shall close this hasty sketch.

"The horse, we may remark, like other large animals, is slow in acquiring maturity, and like them is not very short lived. Some celebrated writers have considered the natural period of his life as about fifty years. This was before the art of shoeing commenced, and may not be far from the truth in those times. If we were to give an opinion on this matter, we should state it as our belief, that he acquires his stature or height at about five years, but obtains his full bulk and strength about the eighth year; and this period, as in most other animals, if multiplied by four, will give somewhat about the period of his natural life: which, without any desire of unnaturally distending, would be from thirty-two to forty; and at the former age we have seen (setting aside the state of his feet) horses capable of a great deal of service. *But what we wish to remark is, that frequent visits to the slaughter house, a useful school, but not much frequented, have led us to observe and conclude, that six arrive there before, to one after, the fourteenth year.*"

